# Threats and Shifts in Microbial Communities of Salars Associated With Direct Lithium Extraction and Brine Reinjection

**DOI:** 10.1002/gch2.70119

**Published:** 2026-09-08

**Authors:** Carolina F. Cubillos, Pablo Aguilar, Cristina Dorador

**Affiliations:** ^1^ Laboratorio de Complejidad Microbiana Instituto Antofagasta y Centro de Bioingeniería y Biotecnología (CeBiB) Universidad de Antofagasta Antofagasta Chile; ^2^ Departamento de Biotecnología Facultad de Ciencias del Mar y Recursos Biológicos Universidad de Antofagasta Antofagasta Chile; ^3^ Centro de Astrofísica y Tecnologías Afines (CATA) Universidad de Antofagasta Antofagasta Chile

**Keywords:** extremophiles, hypersaline environments, lithium, microbial diversity, sustainability

## Abstract

Lithium is a much in‐demand element that is critical for the transition toward low‐carbon energy systems. Beyond hard‐rock mining, large quantities of lithium are extracted from brines of high‐Andean salars (*salares*). The functioning of these microbial ecosystems depends on a delicate equilibrium between mineralogy, water, physical conditions, and microbial life. They are polyextreme environments where life thrives under limiting conditions. In the Salar de Atacama, Chile, lithium is generally extracted through sequential brine evaporation, where lithium is enriched from ∼0.2 to ∼6% w/w. This process has been associated with significant environmental and social consequences, leading to growing interest in implementing new processes, such as direct lithium extraction (DLE), promoted as ‘environmentally friendly’ alternatives. However, this involves the use of chemical reagents and the reinjection of treated brines into the salar, which may have ecological implications that have not yet been fully evaluated. Here, we analyse the main DLE techniques and their potential consequences for microorganisms at community and cellular levels. We argue that DLE technologies, though presented as sustainable alternatives, could potentially have environmental impacts and that current lithium extraction practices continue to conflict with several Sustainable Development Goals.

## Graphical abstract

Lithium for rechargeable batteries is increasingly extracted from brines in high‐Andean salars. Direct Lithium Extraction (DLE) technologies are promoted as sustainable alternatives to conventional evaporation ponds. This work explores how brine modification and reinjection may affect microbial communities and ecosystem functioning, highlighting the need to incorporate microbiological perspectives into sustainability assessments.

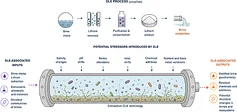

## 1 Introduction

*Salars* are remanent endorheic basins of palaeo‐lakes. They are ecologically fragile and unique ecosystems, yet they play an often‐overlooked but essential role in the global energy transition due to their reserves of lithium [1]. The unusual size of these reserves reflects a conjunction of geological, hydrological, and climatic factors associated with the high‐elevation Andean Plateau, where most of these ecosystems are located [2]. These singularities have resulted in the accumulation of high lithium concentration in the brines of these ecosystems [1]. For instance, the concentrations found in Salar de Atacama, the main worldwide lithium reserve, reach up to 1500 ppm [2]. Chilean salars, together with those from Bolivia and Argentina dominate reserves [3].

The lithium industry is constantly growing, with projections of the total global demand approaching 30 million tonnes by 2050 [4]. Traditionally, brines are pumped (an estimated 185 000 m^3^/day) from the saline crust of salars at depths between 30–50 meters [5]. Lithium is then concentrated *in situ* in sequential evaporation ponds, producing lithium‐enriched brine, which is later used as a reagent for chemical conversion to lithium carbonate [5]. Although the evaporitic process is driven by the high solar radiation of this geographical zone, the conventional lithium concentration technology, or parts of it, cannot be considered a natural phenomenon. The well‐studied Salar de Atacama is an endorheic basin with a hydrological balance of ∼14.9 m^3^ s^−^^1^, where recharge is mainly driven by groundwater infiltration, and discharge occurs almost entirely through evaporation [6]. The pumping of brines to be lithium evaporation ponds results in more than 90% of the brine evaporating at rates of 2.6 mm day^−1^, effectively producing a new hydrological outlet and disrupting the hydrological balance [1]. Given the intensity of lithium evaporation ponds in the area, the southern portion of the Salar de Atacama should be considered as a highly impacted environment. This industrial process greatly increases evaporation rates, reduces natural brine reserves and as such results in permanent water loss, affecting the natural equilibrium of this sensitive ecosystem. This technique is subject to criticism due to the extended (12‐24 months) period required to reach the desired lithium concentration in the evaporitic ponds [5], but also for the environmental impacts affecting the wider ecosystem. These include reduced soil moisture, increased soil temperatures, and a decrease in vegetation, particularly the drought‐resistant tree Chilean algorrobo (*Neltuma chilensis)* [7]. The loss of this plant, which provides key ecosystem services to local communities is just one of many negative impacts of lithium extraction, causing deep concern in indigenous and local communities [8].

Concerns about the sustainability (i.e., meeting the environmental, social, and economic needs of the present, without compromising the ability of future generations to meet their own needs, United Nations, 1987) of conventional lithium extraction methods have led to significant advancements in new technologies (Table 1). Thus, so‐called emerging sustainable Direct Lithium Extraction (DLE) approaches are presented as a technological solution to the environmental impacts produced by the conventional extraction techniques (i.e., brine evaporation). These emerging DLE methods aim to selectively separate lithium ions from brines using adsorbent materials, membranes, ion exchange resins, or electrochemical methods, leading to the production of a lithium‐enriched solution [1]. DLE technologies will not require the use of large‐scale evaporation ponds as they allow brines to be reinjected, therefore resulting reduced impacts on the hydrological balance, and will use renewable energy through the whole process [1]. Thus, they have the potential to minimize the environmental footprint of Li^+^ extraction by mitigating land use, greenhouse gas emissions, and waste generation [9]. However, so‐called techno‐solutions do not fully mitigate the potential damage that these technologies might cause to the ecosystem [10], producing significant effects in other sites beyond the specific extraction site of lithium called ‘off‐sites of lithium production’ [11]. To date, assessments of DLE remain almost exclusively physicochemical, ignoring the ecological and microbial components of salars. There have been no systematic studies addressing how DLE influences the structure, diversity, and functioning of microorganisms. These concerns are fundamental since salars are complex, biodiverse microbial ecosystems that play key roles in greenhouse gas cycling in this geographical zone [12].

**TABLE 1:** Potential microbial disturbances associated with Direct Lithium Extraction.

DLE Technology	Principle	Risk Compound (s)	Potential Microbial Impact	Evidence	Refs.
Liquid‐liquid extraction	Physicochemical separation between immiscible liquid phases	Organophosphorus extracts: tributyl phosphate (TBP) or di‐(2‐ethylhexyl) phosphoric acid (D2EHPA); Ionic liquids (Imidazolium‐ or phosphonium‐based)	Alteration of photosynthetic microorganisms (e.g., thylakoids); disruption of microbial mats; growth inhibition; reduced primary productivity; possible eutrophication processes	Laboratory studies and other systems	[30, 33, 34, 35, 36, 37]
Ion exchange resins	Selective ion exchange and adsorption processes	Release of nanoparticles (e.g., Mn^2+^and Ti^4+^), use of strong acids (HCl and H_2_SO_4_) during regeneration	Alteration of redox balance and pH; potential shifts in microbial community composition	Analogous systems	[39, 40, 42]
Electrodialysis/ Li‐selective membranes	Ion separation driven by an electric potential across selective membranes	Potential release of membrane‐derived compounds (e.g., plasticizers such as phthalates)	Damage to microbial membranes; potential induction of oxidative stress	Laboratory studies	[45]
Nanofiltration	Pressure‐driven size and charge‐based separation	Membrane cleaning agents and surfactants (e.g., EDTA, SDS, Triton X‐100)	Membrane disruption; reduced microbial viability; potential biofilm formation	Laboratory studies	[47, 49, 50]
Electrochemical ion‐pumping technology	Electrochemical insertion/extraction of ions using an electrode	Potential release of electrode‐derived metals (e.g., Ni, Co, Mn oxides)	Membrane damage, cytotoxicity, and selective shifts in microbial community composition and function	Laboratory studies and analogous systems	[51, 52, 54, 55, 56]

Salars are diverse continental aquatic (hypersaline and freshwater) ecosystems that can include lake, lagoon, wetland and salt‐crust habitats. Beyond the microbial taxa that have been the subject of much study, salars host a wide variety of larger organisms, including three charismatic species of flamingos (i.e., *Phoenicoparrus jamesi*, *P. andinus*, and *Phoenicopterus chilensis*), reptiles, birds, fish, camelids (e.g., *Vicugna vicugna* and *Lama guanicoe*), macroinvertebrates, and macrophytes among others [13, 14], many of which are considered endemic or internationally important [15, 16]. Notably, mining activities in the region have already impacted or may affect some species [17, 18]. Although demands for lithium extraction continually grow, the associated ecological risks are largely unknown and effectively underestimated, and current environmental impact assessments only consider conventional evaporation technologies. There is a pressing need to consider new DLE techniques in environmental impact assessments, given that microorganisms—the dominant life forms in these ecosystems [19] are probably among the most sensitive to disturbance.

Studies revealing the importance of microbial life in these systems was key to overturning the established dogma that salars and their desert surroundings were inhospitable and sterile environments. These studies first used traditional microbiological techniques, and later DNA sequencing technologies. As such, it has been possible to describe not only a high diversity of microbial taxa and function but also unique microbial habitats (e.g., microbial mats, stromatolites) present in these ecosystems. In general, the main microbial groups found in salars belong to *Pseudomonadota, Bacteroidota, Firmicutes, Actinobacteriota, Chlorofexota* (e.g., [20, 21]), although their dominance can change depending on the habitats within these ecosystems. Along with the bacterial groups already mentioned, salars host microbial lineages that have recently become important for understanding how life evolved (rare biosphere). Moreover, salars also harbour remarkable eukaryotic and viral assemblages [22, 23]. Microorganisms are key to ecosystem functioning through nutrient recycling and controlling the storage and emission of greenhouse gases. As microorganisms play such key roles in salars, a pressing question is how do human‐made chemicals affect these groups of microorganisms?

The Convention on Biological Diversity (1992) included microorganisms as part of the Earth's biodiversity. This has been corroborated by numerous studies relating to the microbiome of human, different species, and the environment. Therefore, they provide various types of ecosystem services—including supporting, regulating, provisioning, and cultural—according to the categories defined by the Millennium Ecosystem Assessment [24]. Microbial taxa (Bacteria, Archaea, protozoa, fungi) are responsible for ecosystem services: i) supporting and regulating though decomposition, nutrient recycling, climate regulation, water purification, primary production, carbon sequestration, ii) provisioning, including the production of precursors to industrial and pharmaceutical products and iii) cultural, where microorganisms have an important role in education and outreach [25], including new ways to comprehend extinctions, i.e., ‘microdisasters’ [26]. The key role of microorganisms in biogeochemical cycles is well established; however, they are largely absent from environmental regulations or considered when cataloguing biodiversity loss. Considering Earth as a system, it has recently been reported that six of nine planetary boundaries have been exceeded. Changes at the planetary scale forced by humans are causing serious consequences and disequilibrium compared with a ‘Holocene‐like’ interglacial state [27]. As such, salars represent a valuable model to understand microbial life under extreme conditions and how it would be affected by climate change and/or human activities such as mineral (e.g., lithium) extraction. Discussions regarding the biodiversity agenda, including studies about climate change or public policy development rarely include microorganisms (e.g. refs. [28, 29])). Considering the rapid advances in DNA sequencing and understanding of microbial ecology in the last decades, there is an urgent opportunity, and given their importance in ecosystem function, there is an urgent need to include microbial knowledge in our discussions regarding local and global change.

## 2 Potential Impacts on Microbial Life, Function and Habitats

DLE encompasses a range of technologies with distinct mechanisms, and therefore, their environmental impacts should not be considered uniform. The potential impacts discussed in the following section are derived from a combination of laboratory studies and, evidence of analogous systems. Empirical data from salar ecosystems under DLE conditions remain absent.

**FIGURE 1:**
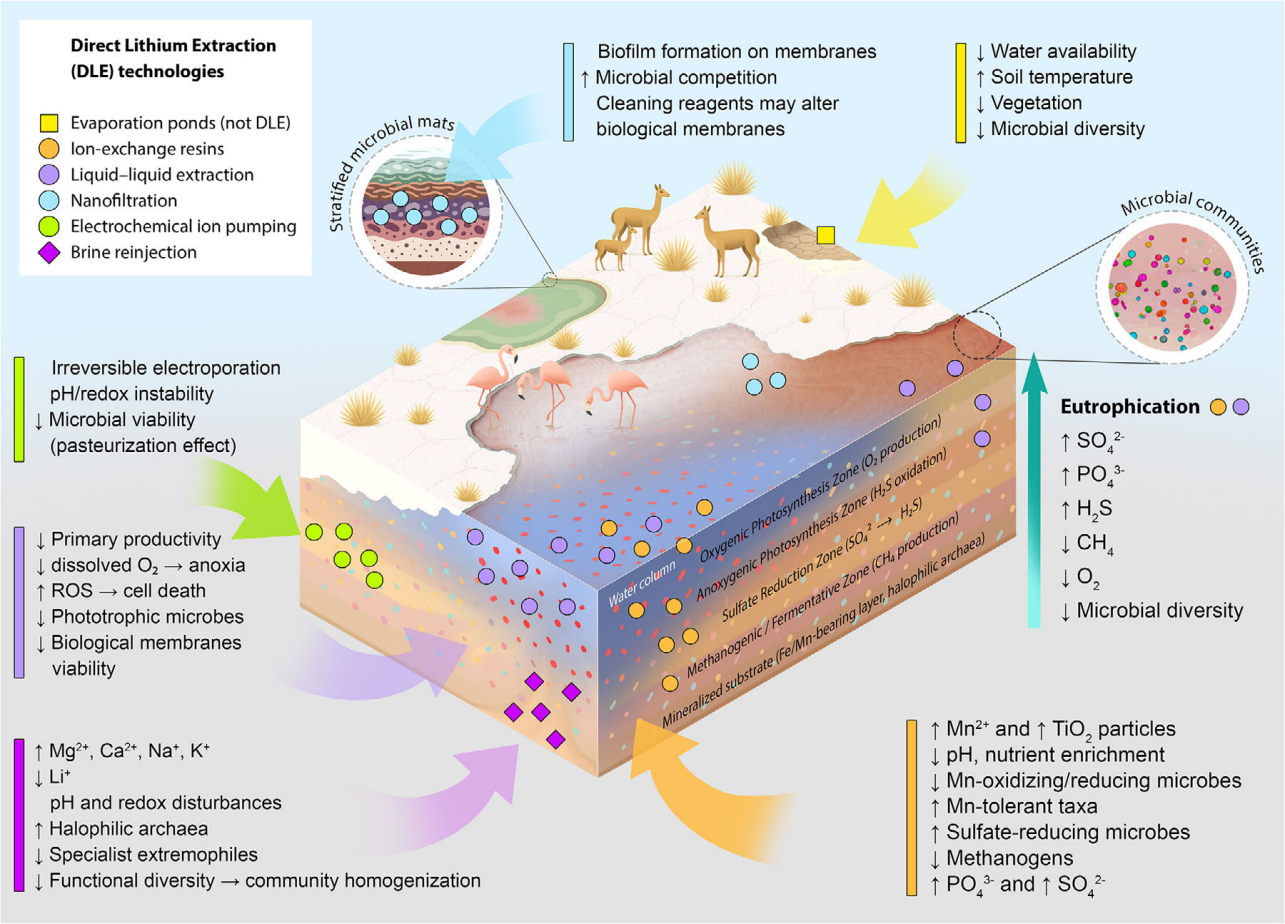
Conceptual representation of potential microbial and geochemical alterations associated with Direct Lithium Extraction (DLE) technologies in salars. Arrows indicate direction and magnitude of physicochemical and biological changes across the microbial mat and water column.

The use of these technologies may affect microbial habitats in different ways (Figure 1). For instance, organophosphate extractants used in lithium and magnesium separation in brines [30] can alter the habitat of an essential biological groups, such as the primary producers, and damage cellular components responsible for photosynthesis (i.e., thylakoids). These groups, composed by cyanobacteria, diatoms, among others, not only generate microhabitats for heterotrophic communities but directly or indirectly fuel metazoan consumers, including emblematic birds such as flamingos. In general, the structure of microbial communities is characterized by the presence of stratified photosynthetic communities called microbial mats, which are highly sensitive to changes in solar radiation (e.g., [31]). They also serve as sensitive indicators of pollution and, in the case of diatoms, as sentinels of hydrological and climatic changes in the Andes [32]. These compounds have been reported in other systems to produce effects such as alterations in lipid metabolism, mitochondrial breakdown, chronic toxicity in fish and invertebrates, as well as reproductive and neurodevelopmental problems in organisms [33]. Specifically for microorganisms, possible scenarios will be evidenced through growth inhibition, overproduction of reactive oxygen species (ROS), and accelerated cell death [34]. Disturbances to these communities caused by the organophosphate extractants may be reflected in biological indicators of ecosystem functioning, such as reduced primary productivity and dissolved oxygen depletion generating eutrophication. Although such damage will not always be immediately evident, one possible consequence could be the decrease of flamingos, associated with the decline of their food resources.

Other solvents associated with DLE technologies are ionic liquids. These are widely promoted as a sustainable option in solvent extraction processes due to their zero gas emissions and potential recyclability, however, their classification as eco‐compatible remains contentious. Their interactions with living organisms are allegedly benign but evidence of significant toxicity exists (e.g., [33, 34, 35, 36]). This contradiction highlights the need to redefine the criteria for a ‘green compound’, incorporating not only physicochemical parameters, but also microbiological assessments. The high solubility of these compounds, which provides the advantage of extracting the ion of interest (i.e., lithium) from liquid solutions (i.e., brines), may lead to deep water contamination, accumulation in aquatic organisms, and persistence over time [35]. With increasing alkyl chain length, these compounds exhibit greater toxicity, manifested as cellular stress and reduced proliferation in prokaryotic cells [36]. Their phospholipid membrane binding site causes cell death in a broad spectrum, ranging from algae and other microorganisms to fish and mammals [37].

Mn‐ and Ti‐ adsorbents resins are considered promising materials due to their high durability in harsh conditions. However, their prolonged use unavoidably poses a risk, namely the release of particles to the environment. Manganese plays a crucial role in cellular stress and in the photosynthesis of phototrophic organisms [38]. The sustained release of Mn‐containing materials could potentially result in significant environmental impacts (e.g., a decrease in pH and an increase in Mn^2+^ solubility), impacting the presence and function of oxidizing and reducing manganese microorganisms. This arises from the fact that chemolithotrophs and phototrophs depend on neutral to alkaline conditions to sustain their oxidative metabolism, while the absence of Mn (III/IV) as an electron acceptor lessens the reducers activity [39], favouring the enrichment of Mn‐tolerant microorganisms. Conversely, the typical dominance of *Pseudomonadota* and *Bacteroidota* in salars would likely be disproportionately favoured in the presence of titanium dioxide particles, negatively impacting other microbial lineages, as observed in agricultural soil with these nanoparticles [40]. As such, artificial changes in microbial structure due to the use of toxic chemical reagents could generate unexpected effects at systemic levels.

Ion exchange resins, mainly organic polymers such as ‐SO_3_H, ‐PO_3_H_2_, and ‐COOH, can release lithium ions. However, resins must be regenerated with acid solutions after the process, generating a residue brine enriched in chlorides, sulphates, and in some cases phosphates [41]. The ionic imbalance in these brines may not be harmless. It could potentially stimulate the growth of sulphate‐reducing communities, increase the production of toxic sulphides, consuming oxygen, and displacing methanogens, altering biogeochemical cycles [42]. Furthermore, phosphate enrichment can result in eutrophication, promoting typical microbial taxa from eutrophic ecosystems, resulting in a loss of functional diversity [43]. Additionally, plasticizers used in electromembranes are not covalently bound to the polymer matrix, and therefore can leach easily into aqueous media. When contaminating plasticizers are released in marine environments, some microorganisms can activate molecular responses, such as expression of degradative esterases to cope with the toxicity of plasticizer side chains [44]. However, this capacity will likely collapse given the extreme ionic complexity of lithium brines as they are up to six times more saline than seawater and include a diverse and enriched ionic composition that extends beyond NaCl. The resilience of salar extremophiles to such synthetic pollutants remains entirely untested. To date, disruption of membrane integrity, oxidative stress, and impairment of energy metabolism in bacteria and eukaryotic cells are the most harmful effects associated with phthalate exposure [45].

The efficiency of nanofiltration technology for lithium recovery is strongly dependent on the ionic composition of brines. It is proposed as an optimal option during post‐processing during the process of lithium extraction [46]. This technology is commonly presented as an environmentally friendly process as additional chemical reagents are not needed [47]. The minimum reagent needed is frequently described during the operation of the process; however, potential environmental impacts may still arise from using some reagents for cleaning or maintaining the membranes due to the risk of them being released to the environment [48]. These compounds may compromise microbial viability by increasing membrane permeability, disruption of the lipid bilayer, and inducing leakage of cellular components, among others [49, 50]. Additionally, the colonization of the membrane by certain microorganisms can lead to biofilm formation [47], which, once the membranes are discarded, may be released into the surrounding environment (e.g., near microbial mats), causing microbial competition.

## 3 Endangered Life in Brines

Brines with elevated lithium concentrations support understudied but diverse communities of halophiles. The application of electrical currents in electrochemical lithium extraction processes could have detrimental to microorganisms. Although the application of highly controlled electric current can promote cell growth and rapid assimilation of nutrients, its biocidal effect has been widely demonstrated [51] and must be considered in natural and polyextreme environments such as the salars. Bacteria and yeast with their peptidoglycan‐rich and chitin/glucans cell walls, respectively, can confer proton‐conducting properties. This feature allows the cell wall to function as a ‘natural Faraday cage’, deflecting electrical current and reducing damage to the plasma membrane [51]. However, a high peptidoglycan concentration does not guarantee higher cell viability. For instance, gram‐positive bacteria, despite their thick peptidoglycan layer, are more sensitive than gram‐negative bacteria, as they lack the outer membrane whose lipid and lipopolysaccharide composition offer additional protection against electric fields. As such, electric current would act as a selective factor affecting the behaviour and fate of microbial cells. Another important effect over microorganisms is that brines, a solution rich in ions, have high electrical conductivity. A significant electrical field generates heating by the Joule effect and therefore could result in irreversible electroporation in microbial cells. Additionally, materials commonly used in lithium‐selective electrodes can release trace metals (i.e., Ni and Co) that produce direct cytotoxic effects, including membrane destabilization and interference with metalloproteins [52]. This can also restructure microbial communities, for instance by increasing the relative abundance of Actinobacteriota taxa and favouring biofilm formation [52, 53]. Furthermore, electrochemical reactions produce drastic changes in pH and oxidation potentials, and thus, the presence of microorganisms in these reactions can affect the membranes in the logarithmic phase of microbial growth, a key stage for cell renewal [54, 55]. This does not consider the impact of the change in polarity required to release ions and obtain a lithium‐enriched solution. Therefore, the combination of electric fields, ionic gradients, and electrochemical by‐products may act as a large‐scale pasteurization‐like system with a high impact on microbial communities, as reported in the leather industry [56].

## 4 A Critical Step That Potentially Affects Salars: Brine Re‐Injection

The following section explores the potential ecological consequences of brine reinjection through a conceptual framework obtained from microbial ecology and geochemistry knowledge.

The re‐injection of ‘spent’ or ‘lithium‐depleted’ brines is repeatedly presented as a mitigation strategy to prevent water depletion in these unique, water‐limited ecosystems and thus, prevent their drying up. In parallel, re‐injection is promoted as a mechanism to ‘eliminate’ the waste generated by direct lithium extraction technologies, under the argument that it returns ‘what belongs to the salar’. However, to date, no studies have been conducted assessing the effects of re‐injection on the diversity, structure, and functioning of microbial communities. Our experience studying the microbial ecology of these high‐elevation ecosystems allows us to suggest that this practice may pose risks of potentially irreversible and large‐scale environmental damage. Earlier activities in two salars (Salar de Lagunillas and Salar de Llamará) included the injection of externally‐sourced water to sustain the water table. Although the full impacts of this practice have not yet been adequately assessed, these salars already show significant negative ecological shifts [57]. One of the arguments in favour of re‐injection of lithium‐depleted brines is that ‘the same water is returned’ to the system; however, multiple aspects have not been considered, extending across physico‐chemical, biological, and ecosystemic levels. Firstly, the reinjected brine differs markedly from the natural one: it is devoid of Li^+^, but it usually enriched in other ions such as Mg^2^^+^, Ca^2^^+^, Na^+^, and K^+^. This is because ion exchange resins and ‘selective’ membranes are not highly specific and can also capture or partially release these cations [58]. Consequently, the Mg/Li ratio would increase, as lithium is retained, altering the geochemistry of the brine. Furthermore, although pH and redox potential changes are inherent to electrochemical or solvent‐based processes, their ability to induce modifications in chemical speciation and promote precipitation in lithium brines has not been systematically studied. However, it is reasonable to anticipate that such alterations would disturb the natural equilibrium of the system. At the biological level ion gradients could affect the composition of extremophile communities. For instance, shifts in microbial communities and competition between methanogens and sulfur‐related microorganisms have been observed in brines in Poland [59]. This finding confirms that not only microbial viability, but also the functioning of key biogeochemical cycles depends on ionic composition. Experimental studies on habitability in brines have demonstrated that the capacity of microorganisms to thrive in a habitat is determined by the combination of ions and cellular physiology, rather than solely by physicochemical variables such as water activity, ionic strength, or chaotropicity [60]. These results reinforce our viewpoint that, although reinjected brines may appear chemically similar to natural brines, being devoid of lithium and enriched in other ions, they may alter the microbial balance and could potentially lead to irreversible ecological consequences.

Lithium enrichment of brines has been reported to play a key role in modulating the microbial community, shifting the composition from halophilic archaea to halotolerant microorganisms [61]. This leads to the following disruptive question: what would happen if, instead of increasing, lithium was to decrease in these systems? A plausible first consequence would be an increase in halophilic archaea to the detriment of lithium‐adapted extremophilic communities, implying the loss of unique lineages. In the short term, this could manifest as changes in relative abundance, ultimately leading to the disappearance of specialists and the proliferation of generalists. The result would be a homogenization of communities, with reduced functional diversity and irreversible alterations in complex structures such as microbial mats and stromatolites. Also, this could have consequences in interactions with other organisms at the microbiome level. If the source of microorganisms is affected, the physiology and other characteristics of plants, animals, and other organisms will also be impacted.

Lastly, although it is often assumed that extremophiles are highly resilient to environmental perturbations, repeated empirical evidence suggests the opposite. Communities from an anthropogenic solar saltern exposed to recurrent fluctuations showed significantly higher robustness than those from natural ecosystems [62]. This confirms that not all hypersaline systems respond uniformly to disturbances. In salars, microbial communities have persisted for millennia, under conditions of relative geochemical stability, which would make them more vulnerable to abrupt disturbances. Repeated discharges of industrially modified brines could not only exceed their resilience, but could also cause permanent damage. As a result, brine re‐injection has the potential to alter the natural gradient, triggering cascading effects from the microbial to the ecosystem level.

The growing interest in DLE technologies highlights the urgent need to critically evaluate their environmental compatibility. Current environmental assessment frameworks associated with DLE technologies rarely incorporate the biological components of salar ecosystems, particularly microbial communities. Therefore, sustainability assessments of these technologies should explicitly incorporate their potential impacts on microbial communities and ecosystem functioning. Any future development aimed at lithium extraction from brines should prioritize minimizing inputs, reducing unintended alterations in brine composition, and avoiding large‐scale perturbations of natural physicochemical gradients. In addition, the implementation of closed‐loop systems with reduced water use, together with more selective extraction approaches, could help limit disturbances to salar environments. Future research should prioritize establishing robust microbial baselines prior to any DLE pilot implementation, followed by controlled experimental studies, *in situ* and long‐term monitoring, and comprehensive assessments to determine how DLE processes and brine reinjection may alter, over time, the biogeochemical and ecological balance of salars, including microbial communities. Until such evidence is available, the assumption that DLE represents a sustainable alternative remains uncertain.

Different DLE technologies, including the re‐injection of modified brines, may lead to similar outcomes, potentially causing chemical alterations in salars that are unlikely to be neutral for microbial communities. In a context where the energy transition cannot be separated from the sustainability of the ecosystems that sustain it, can the energy transition, conceived as a response to the climate crisis, justify the sacrifice of ecosystems that constitute natural laboratories of evolution? The discussion, however, goes beyond strict biology. Recognizing the complexity and fragility of these ecosystems implies rethinking what we mean by ‘green technologies’, and it requires an interdisciplinary approach. This should extend from exploring methods of recycling and recovering lithium from used batteries to the development of new biologically inspired materials to mitigate the growing global demand, or even the search for alternatives capable of sustaining energy storage without greenhouse gas emissions. This work also highlights the absence of regulatory and normative frameworks that recognize environmental damage but ignore the very microbial life that permits life on Earth. This is the only way to anticipate the impacts of the energy transition on such fragile ecosystems. From our perspective, the ecological debts for the recovery of salars—true ‘hotspots’ of complex and fragile biodiversity—are extraordinarily high and unattainable. These high‐altitude wetlands—as well as their microbiome, unique evolutionary heritage, and biological memory of millions of years—will not recover. Therefore, are we sure that our future is worth more than our biological past?

## Funding

This work was supported by ANID/Anillo/ATE240021; ANID/Laboratorios Naturales/NELN250003; ANID/Laboratorios Naturales/NEL2230002; CeBiB AFB240001.

## Conflicts of Interest

The authors declare no conflicts of interest.

## Data Availability

Data sharing not applicable to this article as no datasets were generated or analysed during the current study.
